# Comparative Study of Autogenic and Allogenic Chondrocyte Transplants on Polyethersulfone Scaffolds for Cartilage Regeneration

**DOI:** 10.3390/ijms25169075

**Published:** 2024-08-21

**Authors:** Tomasz Jakutowicz, Monika Wasyłeczko, Maciej Płończak, Cezary Wojciechowski, Andrzej Chwojnowski, Jarosław Czubak

**Affiliations:** 1 Paediatric Orthopaedics and Traumatology Department, Children’s Hospital, Medical University of Warsaw, 02-091 Warsaw, Poland; 2 Nałęcz Institute of Biocybernetics and Biomedical Engineering Polish Academy of Sciences, 02-109 Warsaw, Poland; 3 Department of Orthopedics, Medical Centre of Postgraduate Education, 01-813 Warsaw, Poland; 4 Gruca Teaching Hospital, 05-400 Otwock, Poland; 5 Mazovia Regional Hospital in Siedlce, 08-110 Siedlce, Poland

**Keywords:** autogenic chondrocytes, allogenic chondrocytes, tissue engineering, cartilage regeneration, cartilage implantation, cartilage tissue engineering, scaffold, biomaterials, hyaline cartilage, polyethersulfone scaffold, biomedicine

## Abstract

The aim of this study was to evaluate the chondrogenic potential of chondrocyte transplants cultured in vitro on polyethersulfone (PES) membranes. Forty-eight rabbits (96 knee joints) were used in the project. The synthetic, macro-porous PES membranes were used as scaffolds. Fragments of articular cartilage were harvested from non-weight-bearing areas of the joints of the animals. Chondrocytes were isolated and then cultivated on PES scaffolds for 3 weeks. The animals were divided into four groups. All the lesions in the articular cartilage were full thickness defects. In Group I, autogenic chondrocytes on PES membranes were transplanted into the defect area; in Group II, allogenic chondrocytes on PES membranes were transplanted into the defect area; in Group III, pure PES membranes were transplanted into the defect area; and in Group IV, lesions were left untreated. Half of the animals from each group were terminated after 8 weeks, and the remaining half were terminated 12 weeks postoperatively. The samples underwent macroscopic evaluation using the Brittberg scale and microscopic evaluation using the O’Driscoll scale. The best regeneration was observed in Groups II and I. In Group I, the results were achieved with two surgeries, while in Group II, only one operation was needed. This indicates that allogenic chondrocytes do not require two surgeries, highlighting the importance of further in vivo studies to better understand this advantage. The success of the study and the desired properties of PES scaffolds are attributed mainly to the presence of sulfonic groups in the structure of the material. These groups, similar to chondroitin sulfate, which naturally occurs in hyaline cartilage, likely enable mutual affinity between the scaffold and cells and promote scaffold colonization by the cells.

## 1. Introduction

Articular cartilage covers the ends of bones that make up joints, enabling low-friction motion in the joint. It also plays an important role in load distribution to underlying subchondral bone.

Full-thickness defects of articular cartilage in the knee do not heal spontaneously. Their healing potential is poor due to the lack of blood supply and nervous system [1,2]. Over 200 years ago doctor William Hunter, who was one of the most prominent Scottish physicians and a teacher of anatomy in the 18th century, stated that chondral lesions had been difficult to heal since the times of Hippocrates [1,2].

Cartilage defects may be clinically demonstrated as a general inefficiency of the limbs, intra-articular effusion (usually mild), joint pain, or intra-articular crepitations [1,2,3,4,5,6,7].

Articular cartilage defects are common in the population—according to Curl et al. based on their study group of 31,516 consecutive knee arthroscopies performed for various reasons, articular surface defects were observed in more than 60% of patients [8]. Widuchowski et al. reported about 60% articular cartilage defects, based on their material of 25,124 cases [9]. These defects may progress to osteoarthritis and may require total joint replacement in the following years. Joint replacement surgery is beneficial in the majority of cases, however, there are patients suffering from chronic pain and severe postoperative complications [10]. That is why there is so high a demand for finding a method of treatment that will be as effective as joint arthroplasty, but much less invasive.

Articular cartilage defects comprise a spectrum of disease entities from single, focal defects to advanced degenerative disease of articular (hyaline) cartilage. The cartilage damage is also present in combination with other joint lesions such as meniscal tears. The process of cartilage deterioration mentioned above, involves synovial inflammation [8,9]. Making a diagnosis generally requires magnetic resonance imaging (MRI) to accurately assess the location of specific defects. Depending on the patient’s age, intensity of symptoms, and the size of the lesion there are various methods of nonoperative or surgical treatment. Surgical procedures include debridement/chondroplasty, fixation of unstable fragments, marrow stimulation techniques (microfracture, abrasion chondroplasty, osteochondral drilling), osteochondral autograft (mosaicplasty), osteochondral allograft transplantation, autologous chondrocyte implantation (ACI), and matrix-associated autologous chondrocyte implantation [4,11,12,13]. 

Chondrocyte transplantation may provide better histologic tissue than marrow stimulation and is also useful in the treatment of larger defects. Harvested chondrocytes can be auto- or allogenic. In the lab, chondrocytes are released from the matrix and expanded in culture on scaffolds that are different in their shape, structure, and properties. A second surgery is needed for the transplantation of autogenic chondrocytes [4,12,13,14,15,16,17]. 

There is a growing number of studies on stem cells (from f.e., fat tissue) and their potential in the treatment of cartilage lesions, but scientists all over the world still lack a perfect scaffold adequate for the cells to maintain their phenotypic features and ability to produce an extracellular matrix of articular cartilage [4,13,15].

It is worth mentioning, that when there is no access to a lab for chondrocyte cultivation, or when the funds for the treatment are more limited, the deposition of scaffolds in the defective area, after the marrow stimulation techniques may give reasonable results [2,4,11,12,15,16,17,18,19].

A very promising technique for treating cartilage defects is the use of scaffolds to culture chondrocytes (allo-, autologous) or mesenchymal stem cells (MSCs). The scaffold should have adequate parameters: biocompatibility, appropriate structure, and mechanical properties [15,20,21,22,23,24]. To maintain biocompatibility, a suitable material must be selected. Natural, synthetic polymers or a combination of them (hybrid materials) are used to obtain scaffolds [12,15,22,23,25,26,27,28,29]. Due to the sensitivity of natural polymers to the aqueous environment (hydrolysis), temperature, and pressure, it is difficult to choose a suitable method for obtaining them. In addition, they undergo rapid distortion into a gelatinous form, which does not provide favorable conditions for cell culture. This is related to the fact that the regenerated tissue is a fibrous cartilage, which is susceptible to further damage [12,15,21,22,23,24,25,30,31]. Synthetic polymer membranes are characterized by biocompatibility, good mechanical properties, and various other features that can be used to manufacture membranes [18,31,32]. Some of these are degradable to non-toxic products for the body (for example: poly L-lactic acid, phenyl glycidil ether, polycaprolactone) [18,28,32,33,34,35,36,37]. Unfortunately, their premature degradation causes them to become brittle quickly [32]. An example of a synthetic polymer, with good mechanical properties, that is used in medicine, and tissue engineering is polyethersulfone (PES) [38,39]. It is used in hemodialysis [40,41,42], drug delivery systems (DDSs) [43,44], or as scaffolds for cell culture [31,32]. In previous studies, PES scaffolds, have shown suitable properties for culturing chondrocytes and stem cells [31,32]. They have also been shown to be prone to degradation [31,38].

There is no one good method for the treatment of various articular cartilage lesions. Currently, the most promising methods are ACI and microfracture (MF) using membranes. There are no scaffolds that fulfill completely the desired biomechanical and economical expectations available on the medical market.

The novelty of this study was the use of PES scaffolds for allogenic chondrocyte transplants into the articular cartilage lesions in rabbits. The results were compared to autogenic chondrocyte implantations on a PES scaffold and a control group.

The detailed objectives of this study were:

1. Assessment of the chondrogenic potential of autogenic and allogenic chondrocytes cultured on polyethersulfone membranes transplanted into articular cartilage lesions in rabbits.
2. Comparison of the regenerated tissues between study groups.
3. Evaluation of the stability of the regenerated tissues 8 and 12 weeks after operation within groups. These time points were used because after 8 weeks we should observe regeneration and after 12 weeks we are checking the stability of the regeneration.

The study also used in vitro culture for up to 6 weeks on PES membranes.

## 2. Results

### 2.1. In Vitro Study

#### 2.1.1. Number of Cells and Observation of Chondrocytes with Their Products after Cultivation

Cells obtained after enzymatic isolation were counted using a Bürker chamber. About 1.5 × 10^5^ viable cells were obtained after each isolation. 

**Figure 1:**
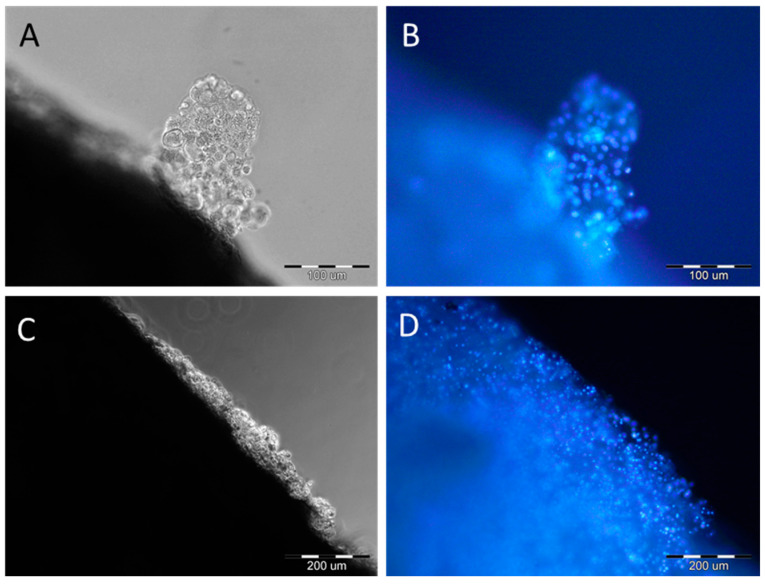
Chondrocytes on the edges of PES scaffolds after six weeks of culture. Samples, before observation, were stained by Hoechst. Scale bars: (**A**,**B**)—100 µm; (**C**,**D**)—200 µm.

After six weeks of culture on scaffolds, images were taken using an inverted microscope with UV light. Figure 1 shows the cells attached to the edge of the membrane along with their staining with Hoechst.

**Figure 2:**
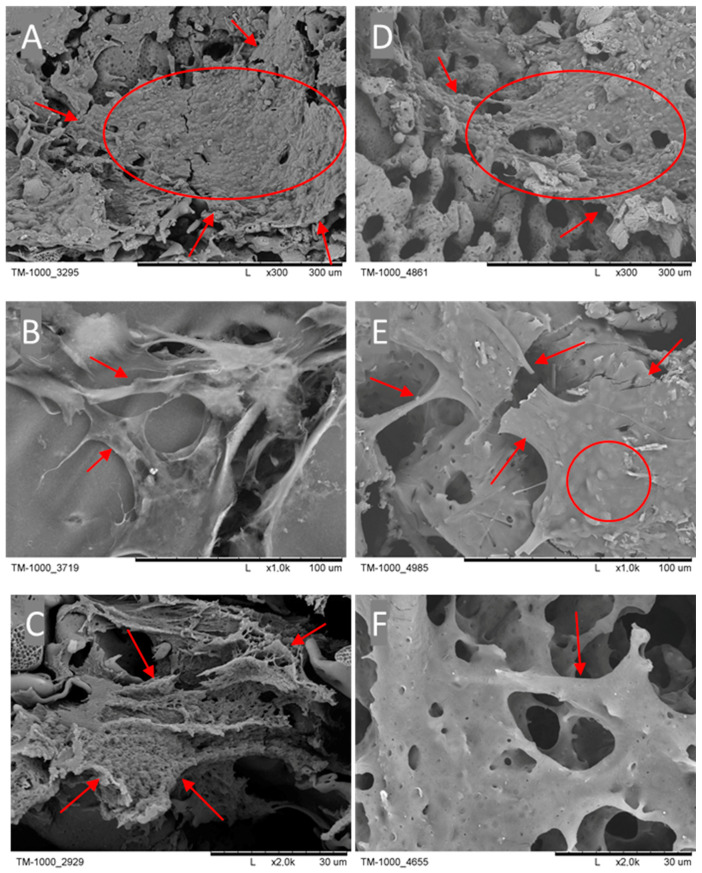
SEM micrographs after six weeks of culture. The red circles and arrows indicate cells with their ECM and newly formed protein. Scale bars: (**A**,**D**)—300 µm; (**B**,**E**)—100 µm; (**C**,**F**)—30 µm.

The presence of cells on the PES membranes was confirmed using a scanning electron microscope (SEM) (Figure 2). Cells with their products, like an intercellular matrix, are noticeable on the PES scaffold, as indicated by the red arrows and circles.

**Figure 3:**
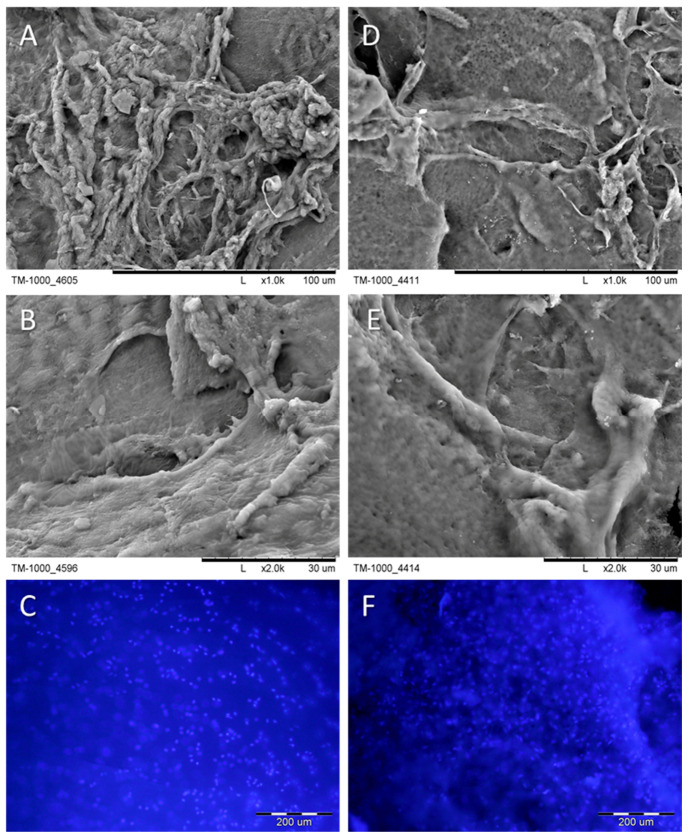
SEM micrographs of native cartilage and recoveries (cells with their products) were obtained from scaffolds after 6 weeks of culture. Samples were stained with Hoechst to visualize the nuclei of cells. (**A**–**C**)—native cartilage; (**D**–**F**)—the recoveries from the PES scaffold. Nuclei staining with Hoechst dye, respectively, for (**C**)—native cartilage, and (**F**)—recoveries from PES scaffold. Scale bars: (**A**,**D**)—100 µm; (**B**,**E**)—30 µm; (**C**,**F**)—200 µm.

The SEM images of recoveries (cells and their products) from scaffolds, native cartilage, and Hoechst dye staining are presented in Figure 3. The surfaces are not smooth (Figure 3D,E), and resemble native cartilage (Figure 3A,B). The presence of cells was confirmed by Hoechst staining where the cells’ nuclei (light blue spots) are visible (Figure 3C,F). The nuclei are not sharp due to their presence in different planes and the irregular surfaces of the samples, making it impossible to capture the correct focus during the experiment.

#### 2.1.2. Elementary Analysis

The nitrogen content was checked before and during cultivation using the elemental analyzer. The control scaffold contains nitrogen because of PVP, which was added to the membrane-forming solution. The content of nitrogen was 0.81% ± 0.12 after 3 weeks of culture and 1.47% ± 0.18 after 6 weeks, respectively. This means that protein content was 5.05% ± 0.73 after 3 weeks of culture, and 9.20% ± 1.13 after 6 weeks, respectively (Table 1). Nitrogen content is an objective and reliable indicator of the amount of protein in a given sample. This proves that the PES scaffold provides a good environment for chondrocyte growth and protein production.

### 2.2. In Vivo Study

#### Microscopic and Macroscopic Evaluation

**Figure 4:**
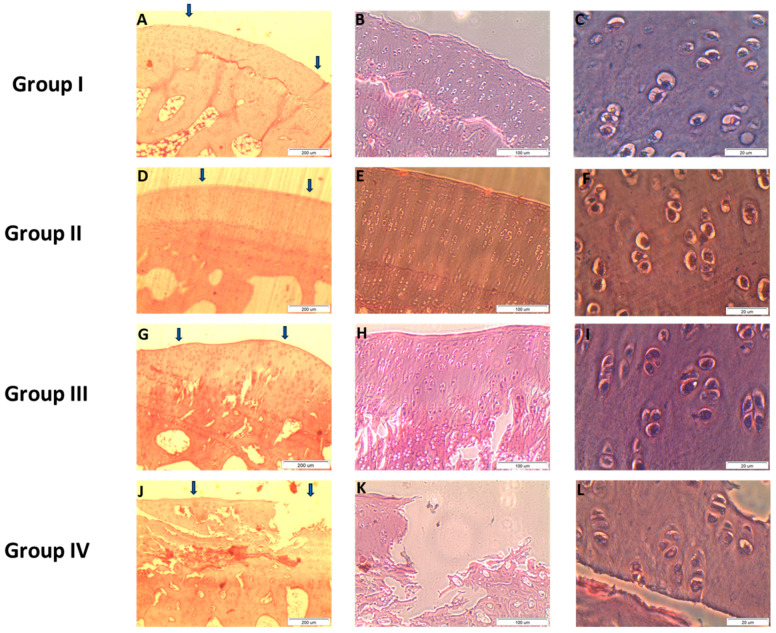
Microscopic images of histological samples of the cartilage of the studied groups (in each row magnification of the photos is increasing from the left to the right side). For group I, II and III, photo (**A**,**D**,**G**), respectively—the defects are bonded at both ends of the graft, group IV is represented by photo (**J**) (arrows indicate the localization of the lesions in all groups). Photos (**A**–**C**): group I after 12 weeks of observation. Photos (**D**–**F**): group II after 12 weeks of observation. Photos (**G**–**I**): Group III after 12 weeks of observation. Photos (**J**–**L**): Group IV after 12 weeks of observation. Scale bars: (**A**,**D**,**G**,**J**)—200 µm; (**B**,**E**,**H**,**K**)—100 µm; (**C**,**F**,**I**,**L**)—20 µm.

In Figure 4, we see among others, the sample from group II after 12 weeks of culture. We can observe that the PES membrane is almost completely broken down. There are no signs of immunological response to the allogenic cartilage.

Figure 4 shows no features of an immune response against cartilage tissue produced by allogeneic chondrocytes. The surface of the regeneration from group I (Figure 4A–C) is smooth and intact, and the thickness of the regenerated tissue is about 100% that of normal cartilage. A similar visualization is shown for group II (Figure 4D–F). The defect is well-integrated and the surface is smooth. Photos for group III (Figure 4G–I) show that the surface of the regenerated tissue is smooth and the cartilage is well integrated. Images for Group IV (Figure 4J–L) after 12 weeks of observation show that the surface of the defect is irregular and there is a lack of integration.

**Figure 5:**
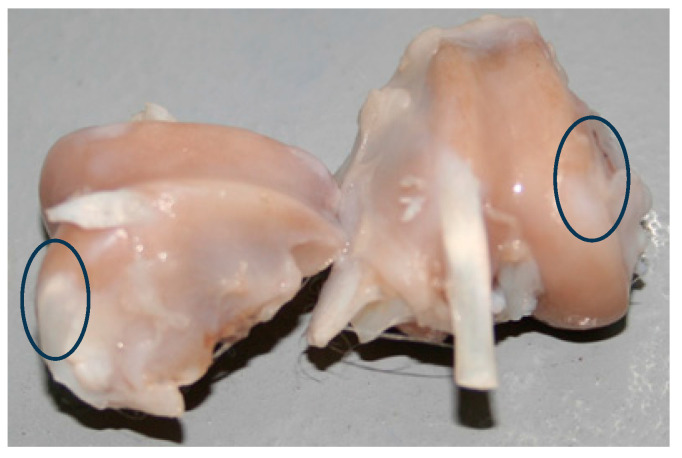
Macroscopic evaluation of the cartilage defect at 12 weeks after implantation for Group II shows complete regeneration.

Figure 5 shows complete regeneration (in circles) after 12 weeks for group II. A comparison of the results of the macroscopic and microscopic evaluations of regeneration within the experimental groups was made according to the Brittberg scale [45] and O’Driscoll scores [46,47] (Table 2).

Based on the above analysis, there was a statistically significant difference in the macroscopic evaluation of articular cartilage regenerates after 8 and 12 weeks in Group II, (*p*-value was 0.047), Group III (*p*-value was 0.027) and Group IV (*p*-value < 0.001).

The next step of the statistical analysis was to check whether a minimum of one group differed statistically significantly from the other groups in terms of scores obtained according to at least one scale (Brittberg and/or O’Driscoll) at 8 and/or 12 weeks postoperatively. The statistical analysis is presented in Table 3. This procedure showed that at least one group differed statistically significantly from the others in terms of the microscopic score obtained (O-Driscoll scale) after a period of 8 weeks postoperatively (*p*-value < 0.001).

Based on data from Table 4, we see that at least one group differs statistically significantly from the others when comparing microscopic evaluation 8 weeks postoperatively (*p*-value < 0.001).

Our next step was to point out which group, or pair of groups, differed from the others when comparing results of the microscopic evaluation 8 weeks postoperatively. We tested this with the Kruskal–Wallis test (Table 3).

Macroscopic assessments after 8 weeks were not statistically significantly different between the groups analyzed (*p*-value = 0.275). This was similar to the macroscopic assessments after 12 weeks (*p*-value = 0.102) and microscopic assessments after 12 weeks (*p*-value = 0.138); the analysis is presented in Table 3.

Multiple comparisons for microscopic evaluation 8 weeks postoperatively showed statistically significant differences between group I and II (*p*-value 0.002), and II and III (*p*-value < 0.001) (Table 4).

## 3. Discussion

The adequate treatment of articular cartilage lesions should be based on the patient’s age, activity level, cause, size and depth of defects, knee alignment, and presence of combined defects. Concomitant defects or malalignment should be treated before surgical articular cartilage repair, or simultaneously [4,13,15,20,24].

ICRS (International Cartilage Repair Society) grade IV lesions do not heal well, and the regenerated tissue is mainly fibrous. Despite the fact that in these types of injuries there is a possibility for bone marrow stem cells to flow into the defect area, the proper cell settlement remains a major issue. Fibrocartilage is biomechanically inferior to hyaline cartilage and eventually results in osteoarthritis [48,49]. What makes a real difference and gives a chance of desired, proper healing in these cases, is the presence of a scaffold. The role of it is to imitate as accurately as possible the structure and function of the articular cartilage extracellular matrix [20,21,22,23,24].

A synthetic PES membrane was chosen for the study because of its suitable properties for chondrocyte culture. It is made of a biocompatible material that has applications in medicine [40,41,42,43,44]. The thickness of the scaffold is 400–500 µm. The scaffold has micro- and macropores. Micropores are 0.01–3 µm in diameter and are used for nutrient supply for cell cultivation and removal of metabolic wastes. Macropores are 40–500 µm, and the vast majority (90–260 µm in diameter) are used for cell settlement. The bottom layer is dense which protects cells and prevents cell loss. The top layer is perforated, allowing cells to enter the membrane. Thus, the structure meets the appropriate requirements for chondrocyte culture [15,18,31,32]. Previous studies have demonstrated the scaffold’s potential to culture rabbit chondrocytes [31] as well as human chondrocytes [32]. Moreover, a previous analysis of an RT-PCR study in an animal model, showed expression of type II procollagen which is typical for hyaline cartilage [31]. Chondrocyte cultivation on collagen membranes gave good results, but the scaffold made of collagen was quickly hydrolyzed, in contrast to PES scaffolds [31].

Lesions 1–2 cm^2^ in low activity patients can be treated with microfractures or arthroscopic debridement as a method of choice, whereas osteochondral allograft transplantation (OAT) is recommended in high activity patients. In patients with high activity, lacking a donor site, and with failures after previous OAT or microfractures, the preferred method will be ACI. Patients with lesions larger than 4 cm^2^ will mostly benefit from ACI conducted with bone grafting if bone loss is present. Focal osteonecrosis, and posttraumatic osteochondral defects larger than 10 cm^2^ are indications for osteochondral allograft transplantation.

The first attempts to cultivate chondrocytes performed in the 1970s in monolayers, showed a significantly reduced concentration of glycosaminoglycan (GAG) and collagen II formation [50].

The mentioned process is, however, reversible when the cell culture is placed in a 3D environment, e.g., in an agarose gel. The cultured cells reveal a chondrocytic rather than a fibroblastic phenotype [51].

Chondrocyte meiotic potential and matrix formation ability decreases over time. Adkisson et al. have shown that proteoglycan concentration was 100 fold higher in cartilage regenerated from donors < 13 years of age than from older patients [52]. Based on that fact, when planning allogenic cartilage transplants, it seems much better to gain the cartilage from juvenile donors. Donors can be minors who have died after severe trauma.

There is no risk of graft rejection because articular cartilage is immunologically privileged thanks to the extracellular matrix [53,54,55].

Mesenchymal stem cells’ (MSCs) transformation into chondrocytes seems promising and is under detailed investigation. It should be stressed that the process is complex and needs certain conditions. The most important ones, among others, are appropriate three-dimensional scaffolds and differentiation factors [56,57,58,59,60]. Transforming growth factor ß—TGFß [59,61,62] and/or bone morphogenic protein—BMP [57,58,63] and glucocorticosteroid are essential for proper cell differentiation and growth.

In our novel study, we compared the outcomes of autogenic and allogenic chondrocyte transplantations on the PES membrane for the first time. We proved that we could achieve good regeneration of the hyaline cartilage lesions by allogenic chondrocyte transplantations. What is more, in allogenic transplantations one operation was needed in comparison to autogenic implantations.

As expected, the best results were in the chondrocyte transplant groups on PES membranes: best in the allogenic group, and slightly inferior in the autogenic. We assume the reason was the fact that in autogenic transplants (group I) we performed two operations—the first for harvesting the cartilage for isolation and cultivation in vitro, and the second operation for implantation of the chondrocytes cultured on PES membranes, in comparison to group II (allogenic transplants), where only one operation was needed (implantation). We assume that the better outcome was because of less surgical stress (less inflammation).

We have obtained surprisingly good results in group III (bare PES scaffolds implanted into lesions) and got even better outcomes in the microscopic evaluations over time (12 vs. 8 weeks postop). We assume that the improvement in the microscopic evaluation over time in group III was due to the migration of the bone marrow stem cells into the scaffold.

Similar research was reported by S.R. Frenkel—he received significantly better results when implanting bare scaffolds (membranes composed mainly of collagen type I) in comparison to leaving empty defects [64]. It has been widely proved that, for proper settlement and proliferation of chondrocytes, a scaffold with 3D structure, imitating the extracellular matrix is essential [64].

The best regeneration was observed in groups II (allogenic chondrocytes transplants on PES membranes) and I (autogenic chondrocytes transplants on PES membranes).

## 4. Materials and Methods

### 4.1. Scaffolds

**Figure 6:**
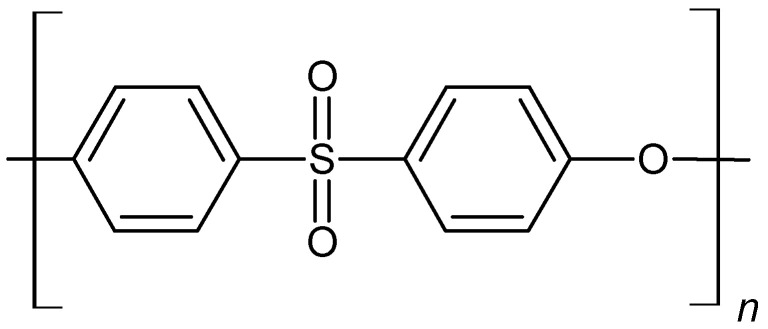
The chemical formula of the polyethersulfone polymer, where n = 50 ÷ 80.

**Figure 7:**
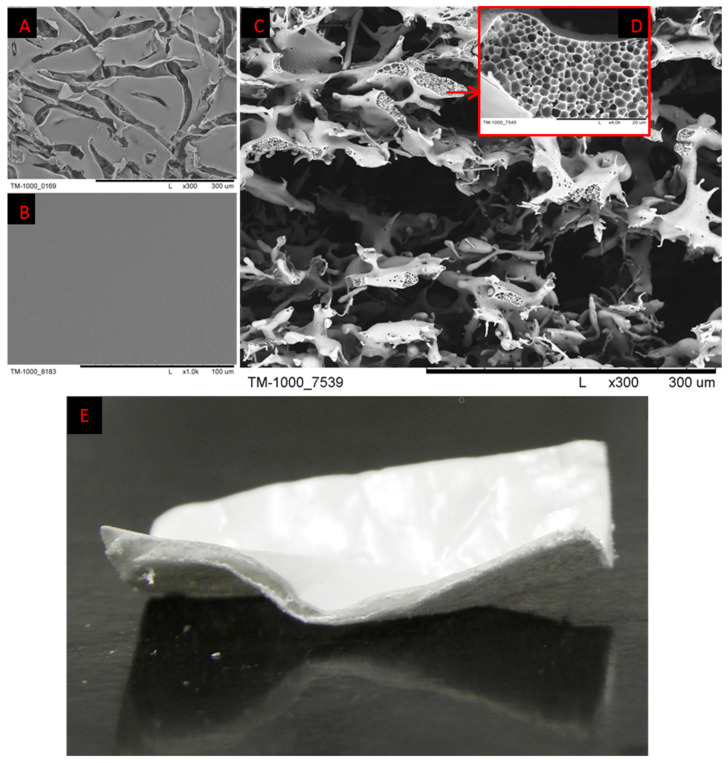
SEM photomicrographs of PES scaffolds: (**A**)—top layer, (**B**)—bottom layer, (**C**)—cross section with macropores, (**D**)—micropores, (**E**)—photo of the scaffold. Scale bars: (**A**,**C**)—300 µm; (**B**)—100 µm; (**D**)—20 µm.

As a scaffold, we used three-dimensional, porous, polyethersulfone (PES) (Figure 6) membranes created at the Nalecz Institute of Biocybernetics and Biomedical Engineering, Polish Academy of Sciences in Warsaw, by the wet phase inversion method (Figure 7).

The PES scaffold was obtained by inversion phase, according to the technique described in previous work [32]. The polymer solution was prepared by dissolving PES and polyvinylpyrrolidone of 10 kDa (PVP) in dimethylformamide (DMF) and N-methylpyrrolidone (NMP) solvents, with constant stirring to obtain 17 wt%. Polymers were mixed in a ratio of 3:1, and solvents were mixed in a proportion of 1:1. Then, the chromatography paper Whatman no. 3 (precursor of macropores) was saturated with a polymer solution. After that, soaked Whatman no. 3 paper was placed on the Whatman no. 1 impregnated with DMF. This composition was immersed in a bath with deionized water for gelation for up to 12 h. To remove cellulose, the membrane was immersed in Schweizer’s reagent. After 4 weeks, scaffolds were placed into a 5% (*v*/*v*) solution of nitric (V) acid to eliminate copper oxide. Then, they were rinsed with deionized water to achieve a neutral reaction. Scaffolds gained were stored in 70% ethanol. Figure 7 presents an SEM image and photo of the PES scaffold. The SEM micrograph of the cross-section of the scaffold (Figure 7D) presents a three-dimensional network of interconnected macropores from 60 to 500 µm diameter. The top layer is perforated with gaps ranging from 10 µm to 1 mm (7A). The bottom layer (Figure 7B) is dense. The addition of PVP affects micropores, which is visible in Figure 7D. The porosity of the membrane is 98.5% and the thickness is about 400–500 µm. The manufactured PES scaffolds were stored in polyethylene bags filled with 70% ethanol. The PES polymer was purchased from BASF company (Frankfurt am Main, Germany) and other reagents were acquired from Sigma-Aldrich (Burlington, MA, USA). Qualitative filter papers (cellulose paper Whatman^®^ no. 1 and no. 3) were purchased from Sigma-Aldrich.

### 4.2. Rabbits

For research purposes, a total number of 48 rabbits (96 knee joints) was used. Group I: 12 animals (24 knee joints), group II: 12 animals (24 knee joints), group III: 8 animals (24 knee joints), group IV: 8 animals (24 knee joints).

Unfortunately, 12 knee joints (from 6 rabbits) were damaged by the pathologist while preparing the specimens. One rabbit did not wake up after anesthesia. One rabbit was excluded due to phlegmon.

The chosen breeds were White New Zealand and White Popielnianski. Animals weighed between 2 and 3.5 kg and were about 4 months old when enrolled in the project. The animal supplier was the Institute of Genetics and Animal Biotechnology of the Polish Academy of Sciences (formerly Institute of Genetics and Animal Breeding of the Polish Academy of Sciences) and Warsaw University of Life Sciences (WULS).The animals were kept under standard environmental conditions: air humidity 25 ± 10%, temperature 21 °C ± 2 °C, 15 air changes per hour, circadian rhythm (light/dark) 12/12 h.

We used rabbits of two different breeds (White New Zealand and White Popielnianski) because at the time we were preparing the study, our animal suppliers, which were the Institute of Genetics and Animal Biotechnology of the Polish Academy of Sciences (formerly Institute of Genetics and Animal Breeding of the Polish Academy of Sciences) and Warsaw University of Life Sciences (WULS) were unable to provide all the rabbits of the same breed needed for the project.

As we did not find any differences in articular cartilage and anatomy in general between the mentioned breeds in the literature, and the inclusion criteria (f.e., age, weight, etc.) were the same for both, White New Zealand and White Popielnianski, we decided to enroll both breeds. All the experimental animals were kept under standard environmental conditions: air humidity 25 ± 10%, temperature 21 °C ± 2 °C, 15 air changes per hour, circadian rhythm (light/dark) 12/12 h. All the procedures were performed by the same surgeon, and all the animals were kept in a dedicated room, in single cages, at the Animal House of the Nencki Institute of Experimental Biology in Warsaw. 

In general, a large amount of research on articular cartilage, especially those with surgical techniques, are performed on rabbit models. The articular cartilage of the rabbit and the human is similar, so the research on rabbits can provide precious information for planning human model studies. The size of the rabbit, as an experimental animal is big enough to perform the surgery (between 2 and 3.5 kg) and not too big to increase the costs of performing the study.

Neither the pathologist nor the biologist were informed about the allocation of the experimental units to control or treatment groups. Only the surgeon and the veterinarian knew which group a particular animal was assigned to.

**Figure 8:**
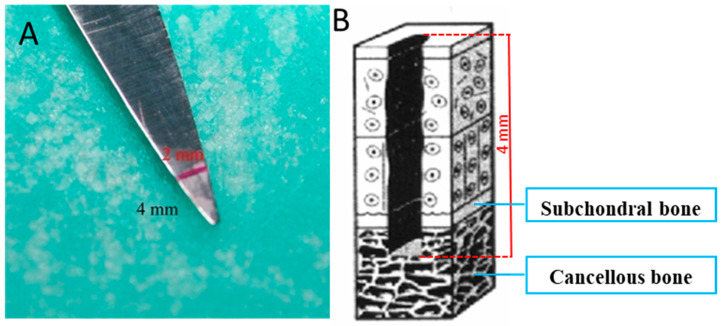
Surgical scissors (**A**) used to generate an ICRS grade 4 full-thickness defect (**B**) in the cartilage. Picture B has been modified according to the literature [65].

We performed standardized, cone-shaped, full-thickness defects (ICRS grade IV) in the articular cartilage with the aid of the triangle-shaped tip of the surgical scissors (Figure 8).

The experimental animals were divided into four groups:

In group I: autogenic chondrocytes on PES membranes were transplanted into the defect area; two surgeries were performed.

In group II: allogenic chondrocytes on PES membranes (chondrocytes taken from group III or IV) were transplanted into the defect area; one operation was performed.

In group III: pure PES membranes were transplanted into the defect area (no chondrocytes transplants); one operation was performed.

In group IV: lesions were left without any treatment (neither chondrocytes nor PES membranes were transplanted); one operation was performed.

In group I animals the first stage operation was articular cartilage fragment harvesting and preparation of chondrocyte cultivation for the planned autologous transplants. The second stage operation in group I was making ICRS IV° lesions and then, subsequently, autologous chondrocyte transplantation. 

In group II animals it was a one stage procedure. Making ICRS IV° lesions and implanting allogenic transplants (cartilage was taken from group III or IV animals).

In group III, implantation of pure PES membranes was performed immediately after harvesting articular cartilage fragments and preparation of ICRS IV° lesions.

In group IV articular cartilage fragments were harvested and ICRS IV° lesions were made. 

The first half of the animals were terminated after 8 weeks and the remaining half were terminated after 12 weeks, postoperatively. Whole knees were cut out and sent in special containers to the pathomorphologist at Warsaw University of Life Sciences. The pathomorphologist prepared the samples for macroscopic and microscopic (O’Driscoll scale) evaluation. The pathologist did not know which group the animal was assigned to.

This research received the approval of the Fourth Local Ethical Commission in Warsaw, for Experiments on Animals, through Opinion No. 13/2010, dated 10 March 2010.

### 4.3. Removal of the Cartilage and Lesion Creation

**Figure 9:**
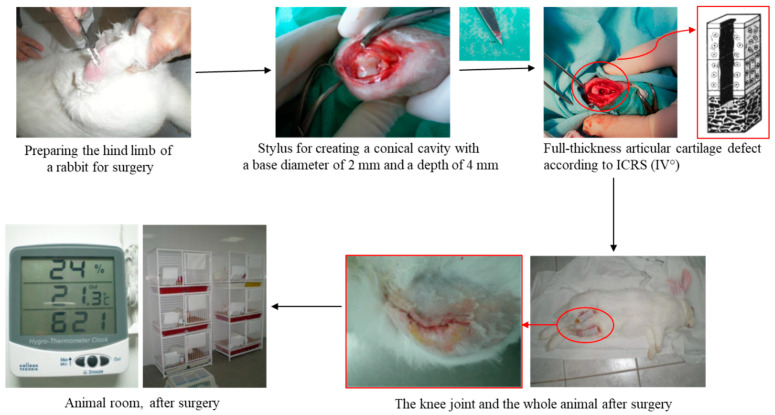
Schema of surgery procedure to create a full-thickness articular defect.

All surgeries were carried out under aseptic conditions in the operating room of the Nencki Institute of Experimental Biology in Warsaw (Figure 9). For general anesthesia of the rabbits, we used ketamine at a dose of 30 mg/kg body weight, which was injected intravenously into the ear vein, and xylazine at a dose of 2 mg/kg body weight—given intramuscularly (into the gluteal muscles). Lignocaine at 1% was used for local anesthesia—it was administered into the knee joint area. The skin around the knees was shaved and disinfected with Hibitane Skin Cleanser. Each rabbit had both knees operated on. We used the medial approach with the subsequent lateral patellar luxation for better insight into the joint. We harvested pieces of articular cartilage from a non-weight bearing area of the joints from experimental animals (Group I, III, IV). We have made full-thickness cartilage defects using triangle-shaped tip of the surgical scissors, with a base diameter of 2 mm and a depth of 4 mm in all groups. (Figure 8). Confirmation of a full-depth defect was the appearance of bleeding from its base. The operation was completed by suturing the wounds in layers, trying to plunge the sutures within the wound edges to reduce the likelihood of them being damaged by the operated animal.

### 4.4. Chondrocyte Isolation

The cartilage fragments collected were transported in sterile tubes in an environment of 0.9% NaCl solution to the Laboratory of the Department of Clinical Cytology of the Centre of Postgraduate Medical Education in Warsaw. In a laminar chamber, using a surgical blade (number 12), the collected cartilage tissue fragments were cut into pieces smaller than 1 mm. The obtained material was washed several times (in sterile Petri dishes) with 0.9% NaCl fluid, and then exposed to a solution of: F-12 (1:1) + GlutaMax TM - I (20 mL), 823 U/mL collagenase type II, 1.15 μL. Dnase at a concentration of 7.2 μg/μL (17.6 units/μg), 10% FBS serum, and antibiotics at 100-fold dilution (Streptomycin and Penicillin). The next step was shaking at 37 °C for 12 h. Then, the samples were filtered through sterile sieves (pore size: 0.7 mm) and centrifuged for 5 min at 1000 rpm at 5 °C. The supernatant was discarded, and the resulting precipitate was resuspended in 1 mL of medium (RPMI) containing 10% FBS and antibiotics (Streptomycin and Penicillin). The isolated cells were counted using a Bürker chamber. Before cell counting, the cells were stained with a 0.5 wt% solution of trypan blue. Sterile 4 mm diameter PES membrane discs were placed in 48-well plates, and an equal number of cells were transferred to the porous side of the scaffold.

### 4.5. Culture of Chondrocytes

Chondrocytes were isolated and then cultivated on PES membranes in the Laboratory of the Centre of Postgraduate Medical Education in Warsaw. Scaffold discs with a diameter of 4 mm were sterilized using ethanol. Then, the sterile scaffolds were rinsed three times with PBS and supplemented medium, and then placed directly into 48-well plates. The chondrocytes were seeded on the scaffold’s perforated layer with a concentration of 1.5 × 10^5^ cells/membrane. The cell cultures were carried out in the incubator at 37 °C and an atmosphere of 5% CO_2_. The medium was exchanged twice a week. The cultivation lasted 3 and 6 weeks at a constant temperature of 37 °C and an atmosphere of 5% CO_2_. After three weeks of cell culture, an appropriate number of PES membranes were transported in sterile tubes in an environment of 0.9% NaCl solution to the animal room for implantation into the knee joints of Group I and II rabbits. The remaining membranes with cells were left for further culture (up to 6 weeks).

### 4.6. Implantation of Chondrocyte Transplants

Bioimplants were implanted into the reopened knee joints of group I, as well as into the first-time opened knee joints of group II rabbits. The transplants were autogenic chondrocytes in group I, and allogeneic chondrocytes in group II. Chondrocytes for allogeneic grafts were obtained from fragments of articular cartilage taken from group III or IV animals. Limb surgery was finished by suturing the wound in layers to reduce the likelihood of suture damage by the operated animals. The animals were reared after surgery in an individual experiment room with electronically controlled temperature, exposure time, and humidity. The animals were also allowed to move around within their cages.

### 4.7. Termination of the Animal Experiment

After a period of 8 or 12 weeks of postoperative observation, the animals were terminated. Whole knee joints were collected and transferred to a histopathologist for preparation of samples and evaluation of articular cartilage regeneration. The material for histological examination was obtained after putting the rabbits to sleep with a lethal dose of Morbital.

### 4.8. SEM Observation

PES scaffolds (without cells) were immersed in ethanol for at least 15 min. They were then placed in liquid nitrogen to break them down into smaller pieces. The sterile scaffolds were rinsed three times with PBS and supplemented medium, and then placed directly into 48-well plates.

Membranes with cells and their products were studied after 6 weeks of culture. The samples were fixed with a solution of 2.5% glutaraldehyde in PBS and were incubated at 4 °C for 1 h. Then, they were placed in a Petri dish and dried at 80 °C.

The samples (with and without cells) were coated with 7 nm of gold using a sputter coater (EMITECH K550X) and observed under an SEM microscope Hitachi TM–1000 with an accelerator voltage of 15 kV. The glutaraldehyde and PBS were acquired from Sigma Aldrich.

### 4.9. Hoechst Staining Procedure

Samples, after 6 weeks of culture on the PES scaffold, were fixed according to Section 4.8. The solution was discarded, and a solution of 100 µL (one drop) of Hoechst dye and 500 µL of PBS was added to each scaffold which was then placed in a 24-well plate. Then, samples were incubated for 20 min and protected from light. They were observed under UV light on a fluorescent microscope (OLYMPUS IX71). The Hoechst 33342 was acquired from Thermo Fisher Scientific, Gibco (Warsaw, Poland) and ethanol solutions (EtOH 70%, and 96%) were provided by Linegal Chemicals (Blizne Łaszczyńskiego, Warsaw, Poland).

### 4.10. Recovery of the Cells and Their Products from PES Scaffolds

PES scaffolds after culture were fixed (described in Section 4.8) and dissolved in dimethylformamide (DMF) with N-methyl-2-pirrolidone (NMP), mixed in a ratio of 1:1. They were kept in the solvent for 2 h with constant stirring. Then, the obtained residue with a new portion of the solvent was centrifuged at 4 °C at 900 rpm for 2 min. The procedure was repeated three times. At the end, the samples were centrifuged three times in deionized water and left to dry. The deionized water (18.2 MΏcm conductivity) was obtained using a Milli-Q apparatus.

### 4.11. Elemental Analysis

Elemental analysis was used to check the PES nitrogen content, before the cultivation (reference samples) and after 6 weeks of chondrocyte culture. The samples were fixed with a solution of 2.5% glutaraldehyde in PBS and incubated at 5 °C for 1 h. Then, they were placed in a Petri dish and dried at 80 °C. The sample prepared was stored in a freezer at −80 °C until further analysis. The control sample was a cell-free membrane incubated in culture medium for 12 h, then washed, dehydrated, and dried as described above. The protein content was assessed by multiplying the determined nitrogen content by a protein conversion factor of 6.25. Two analyses were performed for each sample using a CHNS analyzer (Elementar, Vario EL III).

### 4.12. The Macroscopic Analysis

The obtained regenerations were evaluated macroscopically on a scoring scale according to M. Brittberg (Table 5), where the maximum possible number of points was 12 [45]. The examiner made a visual assessment of the quantitative and qualitative characteristics of the regenerations. The parameters examined were: the degree of filling of the defect, integration with the surrounding cartilage, and the macroscopic appearance of the surface.

### 4.13. The Microscopic Evaluation

Regenerations were evaluated by microscopic examination, according to the O’Driscoll scoring scale (Table 6). The pathomorphologist, using a light microscope, evaluated the quantitative and qualitative characteristics of the regenerations. The parameters examined were the nature of the predominant tissue, structural characteristics, degenerative changes of the cartilage, and subchondral bone reconstruction.

### 4.14. Statistical Analysis

For each group, the mean, standard deviations, medians, and minimum and maximum values were calculated. Using the Kruskal–Wallis test, comparisons were made between each of the regenerate assessments obtained at 8 and 12 weeks separately for each group. The existence of differences between the analyzed groups in macroscopic evaluation after 8 weeks was tested with the Kruskal–Wallis test. If the null hypothesis of equality of distributions in all four groups was rejected, multiple comparisons were made with Bonferroni correction. Each procedure was performed at a statistical significance level of 0.05. We used StatSoftStatistica 2.0 software.

## 5. Conclusions

Despite our access to the most recent research and current knowledge from various countries, perfect chondrogenic differentiation is not achievable with the current differentiation protocols [60].

In precisely performed transplantations of articular cartilage created “in vitro” by allogenic chondrocytes, we do not observe transplant rejections [66,67,68,69,70].

Thanks to modern lab technology and the ability for precise preparation of the bony part of the implant—complete washout of morphogenic elements of blood and bone marrow—some researchers choose xenogeneic materials for the bony part of the transplant and observe no rejection of it [71]. For better consolidation of the cartilage transplant with the subchondral bone it is worth providing Interleukin 1 beta (IL-1β) [72].

In our study, the most similar to articular cartilage were regenerations from group II and I. In group I (autogenic chondrocyte transplants cultured on polyethersulfone (PES) membranes), the results were obtained by two surgeries and in group II (allogenic chondrocytes transplants cultured on PES membranes) only one operation was needed.

In our research, we did not include any kind of immobilization in the postoperative protocol. Animals were able to weight bear as tolerated and we did not set any limitations in the range of movement in the joints to improve the nutrition of the articular cartilage and to promote the production of extracellular matrix, rich in proteoglycans [73,74].

The structure and mechanical properties of PES scaffolds are maintained especially thanks to the presence of sulfonic groups in the structure of the material. Most probably, it enables mutual affinity between scaffold and cells and promotes membrane colonization by cells [31,32].

The best regeneration was observed in Groups II and I. In Group I, the results were achieved with two surgeries, while in Group II, only one operation was needed. This indicates that allogenic chondrocytes do not require two surgeries. Due to this, Group II appears to be more promising. If PES membrane is unavailable, we suggest collagen scaffold, preferably one with smaller pores.

Those with small pores, about 150–250 µm in size, will much better promote the expression and production of type II collagen and aggrecan than ones with bigger pores. It promotes the formation and improves the mechanical properties of the articular cartilage [75].

More research and effort are needed in the field of chondrocyte transplantation techniques to improve them.

## Figures and Tables

**Table 1:** Content of nitrogen which was converted to protein.

Number of Weeks	Content of Nitrogen in Scaffolds (% ± SD)	Content of Protein in Scaffolds (% ± SD)
0	0.48 ± 0.16	0.00
2	0.48 ± 0.21	3.00 ± 1.33
3	0.81 ± 0.12	5.05 ± 0.73
4	1.08 ± 0.21	6.72 ± 1.33
5	1.29 ± 0.09	8.06 ± 0.57
6	1.47 ± 0.18	9.20 ± 1.13

**Table 2:** Comparison of results of macroscopic (Brittberg scale) and microscopic (O-Driscoll scale) evaluation after 8 weeks vs. 12 weeks postoperatively, within groups.

Group	Macroscopic Evaluation Median (Range)			Microscopic Evaluation Median (Range)		
	8 Weeks	12 Weeks	*p*-Value	8 Weeks	12 Weeks	*p*-Value
I	11 (0–11)	11 (0–11)	0.471	16 (0–19)	8.5 (1–19)	0.367
II	11 (8–11)	9 (0–11)	0.047	19 (16–20)	17.5 (0–20)	0.092
III	11 (11–11)	8.5 (0–11)	0.027	11 (3–18)	17 (1–20)	0.187
IV	11 (11–11)	6.5 (0–9)	< 0.001	17.3 (0–20)	13 (0–18)	0.140

**Table 3:** Comparison of results between groups to check if at least one group differs from others according to macroscopic (Brittberg scale) and/or microscopic (O’Driscoll) evaluation after 8 and/or 12 weeks postoperatively.

**Time (Weeks)**	**Macroscopic Evaluation—Median (Range)**				
	**Group I**	**Group II**	**Group III**	**Group IV**	***p*-Value**
8	11 (0–11)	11 (8–11)	11 (11–11)	11 (11–11)	0.275
12	11 (0–11)	9 (0–11)	8.5 (0–11)	6.5 (0–9)	0.102
**Time (Weeks)**	**Microscopic Evaluation—Median (Range)**				
	**Group I**	**Group II**	**Group III**	**Group IV**	***p*-Value**
8	16 (0–19)	19 (16–20)	11 (3–18)	17.3 (0–20)	0.001
12	8.5 (1–19)	17.5 (0–20)	17 (1–20)	13 (0–18)	0.138

**Table 4:** *p*-value for each comparison between groups for the microscopic evaluation 8 weeks postoperatively measured by the Kruskal–Wallis test.

Numbers of Groups Compared	*p*-Value *
I vs. II	0.002
I vs. III	0.027
I vs. IV	0.462
II vs. III	<0.001
II vs. IV	0.379
III vs. IV	0.126

* with respect to Bonferroni correction, the level of statistical significance is 0.008.

**Table 5:** Brittberg Scoring System for Macroscopic Cartilage Evaluation [45].

The Graft Assessment Maximal Score is 12 Points	Criteria	Points
Degree of defect repair	Level with surrounding cartilage	4
	75% repair of defect depth	3
	50% repair of defect depth	2
	25% repair of defect depth	1
	0% repair of defect depth	0
Integration to the border zone	Complete integration with surrounding cartilage	4
	Demarcating border < 1 mm	3
	¾ of graft integrated, ¼ with a notable border > 1 mm	2
	½ of graft integrated with surrounding cartilage, ½ with a notable border > 1 mm	1
	From no contact to ¼ of graft integrated with surrounding cartilage	0
Macroscopic appearance	Intact smooth surface	4
	Fibrillated surface	3
	Small, scattered fissures or cracks	2
	Several, small or few but large fissures	1
	Total degeneration of the grafted area	0

**Table 6:** The O’Driscoll histological articular cartilage repair scale. This was modified according to the literature [46,47].

Category	Subcategory	Characteristic	Score
Nature of predominant tissue	Cellular morphology	Hyaline articular cartilage	4
		Young hyaline cartilage	3
		Incompletely differentiated mesenchyme	2
		Fibrous cartilage	1
		Fibrous tissue or bone	0
Structural characteristics	Surface regularity	Smooth and intact	3
		Superficial horizontal lamination	2
		Fissures 25–100% of the thickness	1
		Severe disruption including fibrillation	0
	Structural integrity	Normal	2
		Slight disruption including cysts	1
		Severe disintegration	0
	Thickness	100% of normal adjacent cartilage	2
		50–99% of normal cartilage	1
		0–50% of normal cartilage	0
	Bonding to the adjacent cartilage	Bonded at both ends of the graft	2
		Bonded at one end or partially at both ends	1
		Not bonded	0
Stage of degenerative cellular changes	Hypocellularity	Normal cellularity	3
		Slight hypocellularity	2
		Moderate hypocellularity	1
		Severe hypocellularity	0
	Degenerative changes	None	2
		Moderate	1
		Significant changes	0
Subchondral bone reconstruction		100%	2
		50–99%	1
		<50%	0

## Data Availability

Data is contained within the article.
